# Letter to the Editor in response to: Microvascular endothelial function following the cessation of long‐term oral contraceptive pill use: A case report

**DOI:** 10.1113/EP091174

**Published:** 2023-04-26

**Authors:** Nathalie V. Kirby, Nina S. Stachenfeld, Lacy M. Alexander

**Affiliations:** ^1^ Noll Laboratory, Department of Kinesiology Pennsylvania State University University Park Pennsylvania USA; ^2^ Department of Obstetrics, Gynecology and Reproductive Sciences Yale School of Medicine New Haven Connecticut USA; ^3^ John B. Pierce Laboratory, Yale University New Haven Connecticut USA

We read the paper by Turner, Stanhewicz, Nielsen, Otis et al. (2023) with great interest, as it addresses an important gap in our understanding of long‐term oral contraceptive pill use and the time course of changes in microvascular function post‐cessation. The authors describe a case study of volitional cessation of long‐term (12 years) oral contraceptive use, in which the 26‐ to 29‐year‐old participant exhibited improved microvascular endothelial function (as indexed by the vasodilatory response to local heating and the nitric oxide‐contribution component) compared with during oral contraceptive use. The authors surmise that vascular and endothelial function are impaired by oral contraceptive use, and thus understanding whether this proposed dysfunction can be recovered upon cessation has considerable implications for women's health across the lifespan. The authors should also be applauded for several aspects of the study itself, including measurements at multiple time points during oral contraceptive use and throughout the menstrual cycle, which captures a more holistic picture of these exogenous and endogenous hormonal effects on the microvasculature. The data collected at 19–22 months post‐cessation address an urgent yet long overlooked question that will be foundational in the design of further prospective experiments. We are writing to encourage this line of investigation and provide additional perspectives for future research.

The augmented cutaneous vascular conductance (CVC) response to local heating (i.e., heating plateau %CVC_max_) was the main basis for the conclusion that endothelial function was substantially increased after the cessation of long‐term oral contraceptive use. However, the authors reported additional outcomes that are interesting to interpret alongside %CVC_max_. Two of three maximal CVC measurements [raw values expressed as red blood cell flux per millimetre of mercury (RBC flux/mmHg)] during oral contraceptive pill use were greater than all post‐cessation values. This mean difference in maximal CVC should not be disregarded and might instead be evidence of increased endothelium‐independent vasodilatation with oral contraceptive pill use, while also explaining the resultant lower %CVC_max_ during the heating plateau. Indeed, this 19% mean reduction post‐cessation aligns with the authors’ more recently published cross‐sectional study (Turner, Stanhewicz, Nielsen & Wong, 2023) showing a mean ∼16% lower maximal CVC in naturally cycling non‐users in the early follicular phase compared with combined oral contraceptive pill users in the placebo pill phase (not statistically significant, *P* = 0.10 in *n* = 9 per group; pills containing norethindrone, *n* = 4; norgestimate, *n* = 1; desogestrel, *n* = 1; drospirenone, *n* = 3). This also aligns with data from Limberg et al. (2016), who observed greater β‐adrenergic‐ and nitroprusside‐mediated vascular conductance in women in the placebo phase of combined oral contraceptive use (norethindrone, *n* = 2; norgestimate, *n* = 3; desogestrel, *n* = 3; drospirenone, *n* = 1; unknown, *n* = 2) compared with non‐users in the early follicular phase and proposed that the vascular smooth muscle is more responsive to nitric oxide in women using oral contraceptives.

The participant in this study was using a combined oral contraceptive containing drospirenone (0.02 mg ethinyl estradiol and 3.0 mg drospirenone), which Turner, Stanhewicz, Nielsen, Otis et al. (2023) specify repeatedly throughout to add important context to their findings. Although this fourth‐generation progestin has been associated with greater thromboembolic risk than other progestins, in vitro studies of drospirenone (Simoncini et al., 2007) and in vivo studies, whereby all (desogestrel or drospirenone; Thompson et al., 2011) or a considerable portion (∼40%; desogestrel or gestodene; John et al., 2000) of the women sampled were using pills containing third‐ and fourth‐generation progestins, also collectively indicate that newer‐generation progestins appear to enhance endothelial nitric oxide synthase expression and concurrently facilitate the beneficial effects of synthetic estradiol on endothelium‐dependent vasodilatation. As would therefore be expected, the participant in the present study consistently demonstrated a lower l‐NAME plateau while using oral contraceptives (∼8%CVC_max_ mean difference) comparable to the mean ∼8%CVC_max_ reduction in the l‐NAME plateau in oral contraceptive users compared with non‐users in the authors’ recent cross‐sectional study (Turner, Stanhewicz, Nielsen & Wong, 2023). Thus, both the mean differences in maximal CVC and l‐NAME plateau values in the present study are relevant when considering the differences observed in %CVC_max_ during the heating plateau in the context of all available evidence.

Independent comparisons of %CVC_max_ at any of the stages of the protocol are also particularly susceptible to issues with interpretation inherent to the main limitation of laser‐Doppler flowmetry; that red blood cell flux is related to, but not necessarily synonymous with, absolute cutaneous blood flow. Moreover, heating plateau %CVC_max_ values are subject to variations in sympathetic, endothelial substrate, and cofactor (e.g., BH4, l‐arginine) influence. Thus, although comparing %CVC_max_ values during the heating plateau has been suggested as a suitable proxy to evaluate endothelial function in the absence of perfusing a pharmacological agent to quantify the percentage of NO contribution directly (Choi et al., 2014), isolating the nitric oxide response to a stimulus most accurately represents the primary aspect of endothelial function (Minson et al., 2001). Based on percentage NO contribution values from the present study (mean ∼70 and ∼60% NO contribution during and post‐cessation of oral contraceptive use, respectively) and in the authors’ more recent work, whereby the percentage NO contribution in oral contraceptive pill users was greater than that in non‐users (∼74 and ∼52% NO contribution, respectively; Turner, Stanhewicz, Nielsen & Wong, 2023), the local vasodilatory response might exhibit an underlying positive shift towards NO‐dependence during oral contraceptive use. These points considered, changes in %CVC_max_ during the heating plateau alone may not provide conclusive evidence of enhanced endothelial and/or vascular function following cessation of oral contraceptive pill use, and we believe that there is ample space to further explore this hypothesis.

Turner, Stanhewicz, Nielsen, Otis et al. (2023) state that ‘[previous] findings suggest that [oral contraceptive pill] use at any time can impact future cardiovascular health (Lee et al., 2022)’. Indeed, Lee et al. (2022) observed that previous oral contraceptive use was associated with an increased risk for hypertension later in life (probably attributable to the sympathetic and resultant blood‐pressure‐modulating effects of older oral contraceptives). Conversely, epidemiological data in post‐menopausal women indicate that previous oral contraceptive use might also protect against atherosclerosis, a disease closely related to endothelial and vascular function (Shufelt & Bairey Merz, 2009; oral contraceptive types/dosages unknown). Although data are sparse and there are important socio‐economic considerations when interpreting these epidemiological findings, this provides additional, albeit indirect, evidence for the beneficial effects of oral contraceptives on the nitric oxide response and smooth muscle function discussed above. This sentiment is also supported by the exploratory subgroup analysis of oral contraceptive users and non‐users in the authors’ recent publication (Turner, Stanhewicz, Nielsen & Wong, 2023), whereby mean differences in all measured outcomes, including those that were not statistically significant, were directionally in accord with oral contraceptive use having beneficial vascular effects. It is worth noting, however, that these long‐term epidemiological data in postmenopausal women and recent cross‐sectional study findings by Turner, Stanhewicz, Nielsen & Wong (2023) indicating probable positive vascular effects contrast with the increased risk of adverse cardiovascular events observed in those actively using oral contraceptives (Shufelt & Bairey Merz, 2009). This highlights the importance of distinguishing between the unique work of Turner, Stanhewicz, Nielsen, Otis et al. (2023) and most previous literature on the topic. Their focus on the transitional period of oral contraceptive cessation provides a particularly interesting model to investigate the ‘missing piece’ in our understanding of the long‐term impact of exogenous hormone exposure on vascular function not explained by previous oral contraceptive intervention studies or cross‐sectional studies.

Newer‐generation progestins might also mitigate the increase in hypertension risk traditionally associated with oral contraceptive pills, because they instead appear to decrease blood pressure (drospirenone; Shufelt & Bairey Merz, 2009), which makes differentiating between progestin types even more important for future research addressing physiological mechanisms underpinning cardiovascular health indices. Although there might be independent effects of the type of progestin contained in different oral contraceptives, the >60 brands of oral contraceptives available in various dose and progestin‐type combinations are also likely to modulate vascular responses (Williams & MacDonald, 2021). In particular, given that conclusive evidence on long‐term vascular health outcomes associated with chronic exposure to specific dose and progestin‐type combinations is currently lacking, it is worth considering that oral contraceptive pills are arguably the most accessible form of hormonal contraception and can provide a wealth of other positive health benefits (prevention of unwanted pregnancy, reduced risk of ovarian cancer, first‐line treatment for reproductive diseases such as endometriosis and polycystic ovary syndrome) and quality of life benefits (reduced acne, management of menorrhagia, comfort during exercise and daily life). We, therefore, caution against over‐generalizing findings from a single participant to all oral contraceptive users. However, we believe that Turner, Stanhewicz, Nielsen, Otis et al. (2023) have exposed an important research gap that will spur future prospective studies and provide valuable evidence to aid women and healthcare providers in making better‐informed decisions when balancing the positive and negative effects associated with specific dose and progestin‐type combinations.

We close this letter by acknowledging the importance of the authors’ work. As noted by Turner, Stanhewicz, Nielsen, Otis et al. (2023), 75%–85% of women globally use oral contraceptives at some point during their premenopausal years, yet the mechanisms underpinning modulated vascular function with cessation of long‐term use have been largely unexplored. This case report provides important hypothesis‐building data to support the additional investigation of this topic, which we commend the authors for highlighting and strongly believe is deserving of further attention.

## AUTHOR CONTRIBUTIONS

Nathalie V. Kirby drafted the manuscript; all authors provided critical edits and revisions and approved the final version of the manuscript.

## CONFLICT OF INTEREST

The authors declare no conflicts of interest, financial or otherwise.

## FUNDING INFORMATION

None.
